# Nuclear diversity in Charophyceae revealed by fluorescence staining and its implications for genome size estimation

**DOI:** 10.1007/s00425-026-05085-w

**Published:** 2026-08-28

**Authors:** Julien Böhm, Julia Peters, Klaus Herburger, Hendrik Schubert

**Affiliations:** 1 Institute of Biosciences, Department Aquatic Ecology, University of Rostock, Albert-Einstein-Straße 3, Rostock, Germany; 2 Institute of Biosciences, Department Cell Biology of Phototrophic Marine Organisms, University of Rostock, Albert-Einstein-Straße 3, Rostock, Germany

**Keywords:** Characeae, Fluorescence microscopy, Flow cytometry, Genome size, Nuclei, Streptophyta, Transmission electron microscopy

## Abstract

**Main conclusion:**

**We established a protocol for reliable nuclear visualization in Charophyceae, revealed diverse nuclear organization across cell types and species, and identified suitable cells for genome size estimation via flow cytometry.**

**Abstract:**

Charophyceae are multicellular green algae closely related to land plants and are established model systems for understanding plant evolution. Yet key cellular parameters like genome size remain poorly characterized. We combined fluorescence microscopy, transmission electron microscopy (TEM), and flow cytometry to characterize nuclear diversity across cell types and species of Characeae and to identify a cell type suitable for genome size estimation. Among three DNA-intercalating fluorochromes, propidium iodide labeled nuclei most reliably. Nuclear morphology varied widely across cell types: mononucleated cells were found in vegetative apical cells, the coronula of oogonia, and spermatogenous filaments of antheridia, whereas multinucleation predominated in other tissues, e.g., cortical cells, spine cells, stipulodes and rhizoids. Nuclei in *Chara hispida* showed a significant gradient in cross-sectional area along the thallus axis. In the apical internodes, nuclei were larger and more heterogeneous, whereas in the basal internodes they were smaller and more uniform, which is consistent with possible endopolyploidy. TEM confirmed the nuclear identity of crescent-shaped structures. Relatively large nuclei were found in rhizoids and spine cells. Only *Sphaerochara canadensis* showed an organized nuclei pattern. Whole-thallus preparations did not yield a defined nuclear peak by flow cytometry, but antheridia of *Chara tomentosa* produced a sharp peak, from which a genome size of 5.32 pg (1C) was estimated. The protocol established here provides a simple, reproducible framework for visualizing nuclei and estimating genome size in Charophyceae, and helps address longstanding questions in this group, such as the mechanisms and functions of multinucleation, the site of meiosis, and genome evolution.

**Supplementary Information:**

The online version contains supplementary material available at https://doi.org/10.1007/s00425-026-05085-w.

## Introduction

Charophyceae (Streptophyta) are aquatic green algae that form a multicellular thallus with clear cellular organization, including differentiated nodes, internodes, lateral branches, and a rhizoid system (Schubert et al. 2024; Fig. S1). Owing to their morphological complexity and early phylogenetic analyses, Charophyceae were long regarded as the closest extant relatives of land plants (Karol et al. 2001; McCourt et al. 2004). However, phylogenomic evidence now supports the Zygnematophyceae in this role (Wodniok et al. 2011; Timme et al. 2012; Wickett et al. 2014; Gitzendanner et al. 2018; Hess et al. 2022). Nevertheless, recent molecular insights have reemphasized their evolutionary importance. For example, the genome of *Chara braunii* C.C. Gmelin revealed key similarities with embryophytes such as phragmoplast formation, supporting their relevance for understanding the evolutionary transition from aquatic algae to terrestrial plants (Nishiyama et al. 2018).

Despite this renewed interest, essential cellular parameters like genome size remain largely unresolved in Charophyceae. Genome size is a fundamental biological trait that influences cell size, division rates, metabolism, developmental rate, and overall body size (Elliott and Gregory 2015). In addition, genome size determination is a crucial prerequisite for whole-genome sequencing, comparative genomics and taxonomic treatments (Pflug et al. 2020). Yet a very limited number of studies report genome sizes for Charophyceae, for example by using staining via Schiff’s reagent and photometry of male gametangia (antheridia) (Shen 1967a; Maszewski and Kołodziejczyk 1991). Flow cytometry, now widely used for genome size estimation, offers a faster and more scalable alternative by staining nuclei with DNA-intercalating dyes and quantifying fluorescence intensity (Doležel et al. 1998). In preliminary experiments with *Chara tomentosa* L. (see Fig. 10a, d) and further *Chara* species (data not shown), however, we were unable to isolate a clearly defined nuclear population by using whole thalli, preventing reliable genome size estimation. This was surprising as flow cytometry allowed for estimating the genome size of *Chara braunii* in an earlier study (Nishiyama et al. 2018). On the other hand, in the same study, the reported genome size deviated slightly from the value obtained through sequencing.

Such technical difficulties are likely rooted in the unique cellular architecture of Charophyceae: cells are often multinucleated, and their nuclei vary in size, shape, and possibly DNA content. Studies from the late 19th and early 20th century revealed a remarkable diversity of nuclear forms, ranging from round, oval and crescent-shaped to elongated and thread-like (Kaiser 1896; Hasitschka-Jenschke 1960; Shen 1967b) and the nuclear identity of round-to-oval forms was later confirmed by transmission electron microscopy (TEM) in *Chara contraria* A. Braun ex Kütz. (Vouilloud et al. 2007). Further observations of endopolyploidization and intercellular differences in nuclear content suggest that Charophyceae possess highly dynamic nuclear systems (Hasitschka-Jenschke 1960; Shen 1967b). Despite this, fluorescence-based and ultrastructural studies of nuclear morphology are scarce, hampering a deeper understanding of nuclei organization and genome size. Adding to this complexity and knowledge gaps, the site of meiosis in Charophyceae remains uncertain. It has been suggested that meiosis occurs during oospore germination (Oehlkers 1916), gamete formation (Tuttle 1924, 1926), or at a specific node of the protonemal branchlet (Gonçalves da Cunha 1936, 1942), as reviewed in Schubert et al. (2024). The  conventional interpretation of the life cycle assumes a haploid plant with zygotic meiosis (Haig 2010; Delwiche and Cooper 2015; Schubert et al. 2024). Resolving these fundamental questions relies heavily on a clear understanding of nuclear organization, dynamics and genome size.

This study therefore aims to (i) establish and evaluate a set of DNA dyes and preparation techniques for the reliable visualization and identification of nuclei in Charophyceae using fluorescence microscopy; (ii) document nuclear morphology and size across cell types, internodal position, and species, with TEM confirmation of unusual nuclear shapes; and (iii) identify a homogeneous cell type that can serve as the basis for genome-size determination by flow cytometry. In doing so, we seek to lay the methodological foundation for future genomic and cytological research in this morphologically complex yet evolutionarily pivotal group.

## Materials and methods

### Collection and cultivation of material

Various Characeae species were collected at different locations (Table 1). Species were identified based on the determination key presented by van de Weyer et al. (2024). Algae were then either used directly for preparations or first cultured as described in Böhm et al. (2025). Images and/or herbarium specimens of the species used are stored at the University of Rostock (ROS) and are available upon request.

**Table 1:** Used Characeae species with sampling site and date. Corticated species are indicated by an asterisk (*)

Species	Site	Coordinates	Date
*Chara aculeolata* *	Lake Badesee Apetlon, Austria	47°47′06.6"N 16°50′14.5"E	05.05.2023
*Chara braunii*	Lake Finjasjön, Sweden	56°08′11.0"N 13°40′32.1"E	19.07.2024
*Chara hispida* *	Lalendorf-Langhagen, Germany	53°40′33.2"N 12°25′58.2"E	31.05.2023
*Chara* sp.	–	–	–
*Chara papillosa* *	–	–	–
*Chara tomentosa* *	Lalendorf-Langhagen, Germany	53°40′35.2"N 12°26′14.1"E	06.06.2024
*Lamprothamnium papulosum*	Beach lake, Cyprus	34°36′51.1"N 32°55′35.4"E	28.04.2024
*Nitella opaca*	Lalendorf-Langhagen, Germany	53°40′34.6"N 12°25′60.0"E	06.06.2024
*Sphaerochara canadensis*	Lake Torneträsk, Sweden	68°21′21.1"N 18°50′27.1"E	25.08.2023
*Tolypella nidifica*	Boflagen, Sweden	57°55′01.4"N 16°33′54.9"E	18.07.2024

## Fluorescence microscopy

Subsamples of corticated species were pretreated with 5 N hydrochloric acid for 10 min to enhance cell-wall permeability, were then rinsed thoroughly with distilled water, and placed in micro reaction tubes containing 1800 µL of phosphate-buffered saline (PBS; 0.1 M, pH 7.4). The respective DNA-intercalating dye was added under dark conditions to visualize the nuclei: 9.9 µM propidium iodide (PI) for 10 min; 3.3 µM 4′,6-diamidino-2-phenylindole (DAPI) for 20 min or 5.5 µM SYTOX Green dye for 5 min. Final dye concentrations refer to the total staining-solution volume. After the incubation period, samples were washed in 1800 µL PBS, cut into 2-cm long sections, mounted in PBS and imaged on a digital fluorescence microscope (BZ-X, Keyence, Neu-Isenburg, Germany) equipped with Fluorite 10×, 20× and 40× LD PH objectives and BZ-X filter cubes DAPI (DAPI; Ex.: 352–402 nm; Em.: 417–477 nm), GFP (SYTOX Green; Ex.: 457–487 nm; Em.: 502–538 nm), and TRITC (PI; Ex.: 532–554 nm; Em.: 570–613 nm). Images were captured with BZ-X800 Viewer software. For preparation of antheridia, the shield cells were punctured with a razor blade to expose spermatogenous filaments.

## Transmission electron microscopy

For fixation and contrasting, samples were fixed using 2% glutaraldehyde in 75 mM sodium cacodylate buffer, pH 7, followed by incubation in 1% O_s_O_4_ overnight according to Quader (1984) and Holzinger et al. (2009). For dehydration a series of graded acetone concentrations (30–100%) was used. Samples were embedded in Spurr’s epoxy resin and sectioned with an ultramicrotome (Ultracut E, Leica-Reichert-Jung, Nußloch, Germany), counterstained with 2% uranyl acetate followed by lead citrate. TEM was performed with a 906 E TEM (LEO; Zeiss, Oberkochen, Germany) at 100 kV equipped with a Wide-angle Dual Speed 2 K-CCD camera from Tröndle Restlichtverstärkersysteme (TRS; Moorenweis, Germany) operated by the software ImageSP-Professional + Panorama (MIA) also from TRS.

## Quantification of nuclear cross-sectional area

Nuclear cross-sectional areas were quantified in fluorescence images of *Chara hispida* L. using the thresholding and analyze particles tools of Fiji ImageJ (Schindelin et al. 2012), with a size filter of 30–200 µm^2^ to exclude debris and blurred nuclei and circularity set to 0.00–1.00. Misidentified objects (overlapping or partial nuclei) were corrected manually using the ROI Manager. For each of six individuals, one image per internodal position (basal, medial, apical) was acquired, and 50–100 nuclei per image were measured. The 50–100 measurements within each image were summarized by their median (as an indicator for nuclear size) and by the range of their areas (as an indicator of size and shape diversity). These per-image summary statistics were then used for further analysis. Two-dimensional cross-sectional area is an indirect proxy for nuclear volume and should not be interpreted as a direct measurement of DNA content.

## Flow cytometry

Flow cytometry was performed using a CytoFLEX flow cytometer (Beckman Coulter GmbH, Krefeld, Germany) following a previously optimized protocol with modifications (Doležel et al. 1989). For *Chara tomentosa*, samples consisted either of a whole thallus (including all cell types and gametangia) or of approximately 50 antheridia per preparation. Leaves of *Secale cereale* cv. Dankowskie served as an internal standard (Doležel et al. 1998). Sample material was chopped with a razor blade in a Petri dish containing 900 µL of lysis buffer (LB01). The resulting suspension was passed through a 70-µm filter (ClearLine, Dominique Dutscher SAS, Bernolsheim, France), and the filter was rinsed with an additional 900 µL of LB01 buffer. Subsequently, 18 µL of PI was added, yielding a final concentration of 9.9 µM; the sample was vortexed briefly, and incubated in the dark on ice for 10 min. Approximately 10,000 events were recorded per sample. PI fluorescence was detected in the phycoerythrin area (PE-A) channel, and side scatter area (SSC-A) was recorded for quality control. Instrument settings were: SSC gain 63, PE gain 30 (both on a 1–3000 range), and a threshold of 25,000. The resulting.fcs files were imported into R (R Core Team 2024) using the flowCore package (Ellis et al. 2024). Major fluorescence peaks were identified by applying a Gaussian smoothing kernel (σ = 5, window size = 15) to the binned fluorescence count data. Peaks were defined as local maxima exceeding a threshold of 30 counts.

## Statistical evaluation and data visualization

The statistical analysis, data processing, and graphical representation of the data were performed in R (R Core Team 2024), using the packages dplyr (Wickham et al. 2014), tidyr (Wickham et al. 2025), tibble (Müller and Wickham 2026), car (Fox and Weisberg 2019), PMCMRplus (Pohlert 2024), ggplot2 (Wickham 2016), ggthemes (Arnold 2012), ggpubr (Kassambara 2016), and patchwork (Pedersen 2025). Normality of nuclear-area data was assessed using Shapiro–Wilk tests, followed by Levene’s test for homogeneity of variances. As not all groups met the assumptions of normality and/or homogeneity of variances, differences among internodal positions were tested using the Friedman rank-sum test and the effect size was reported as Kendall’s *W*, followed by exact all-pairs comparisons of Friedman-type ranked data (Eisinga et al. 2017), with *P* values adjusted by Benjamini–Hochberg procedure. Statistical significance was considered at *P* < 0.05.

## Results

**Fig. 1:**
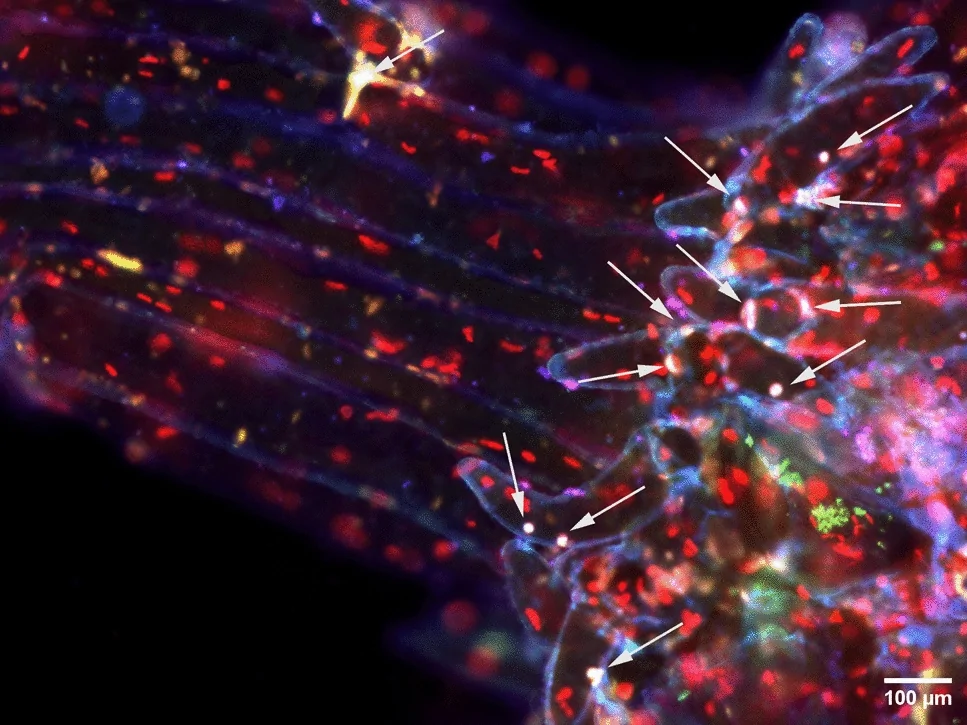
Overlay image of *Chara hispida* stained with PI, DAPI, and SYTOX Green. Red areas indicate exclusive staining with PI, blue with DAPI, and green with SYTOX Green. White areas represent regions stained by all three dyes (arrows)

Comparing the fluorescent dyes PI, SYTOX Green, and DAPI revealed that PI produced the clearest results, with well-defined nuclear structures (Fig. 1 and Fig. S2). Staining with SYTOX Green and DAPI labeled substantially fewer nuclei compared to PI, and a more diffuse background fluorescence was observed. Staining with all three dyes was most successful in the stipulodes (Fig. 1 and Fig. S2).

**Fig. 2:**
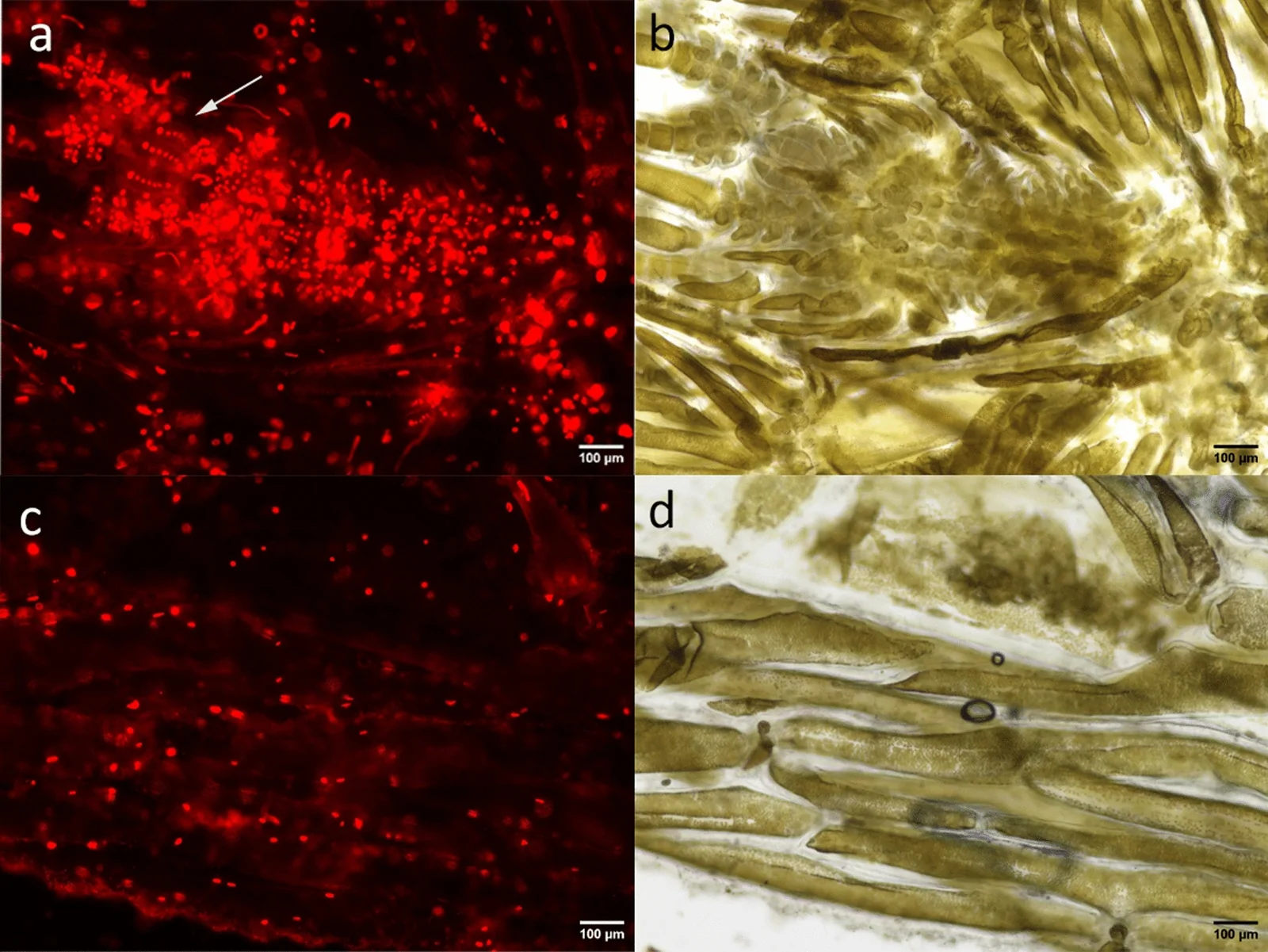
Fluorescence and light microscopic images of apical and basal parts of PI-stained *Chara hispida* cells. **a** Fluorescence image of the apical region. The white arrow indicates the apical initial cells. **b** Light microscopic image of the apical region. **c** Fluorescence image of the basal internode. **d** Light microscopic image of the basal internode

**Fig. 3:**
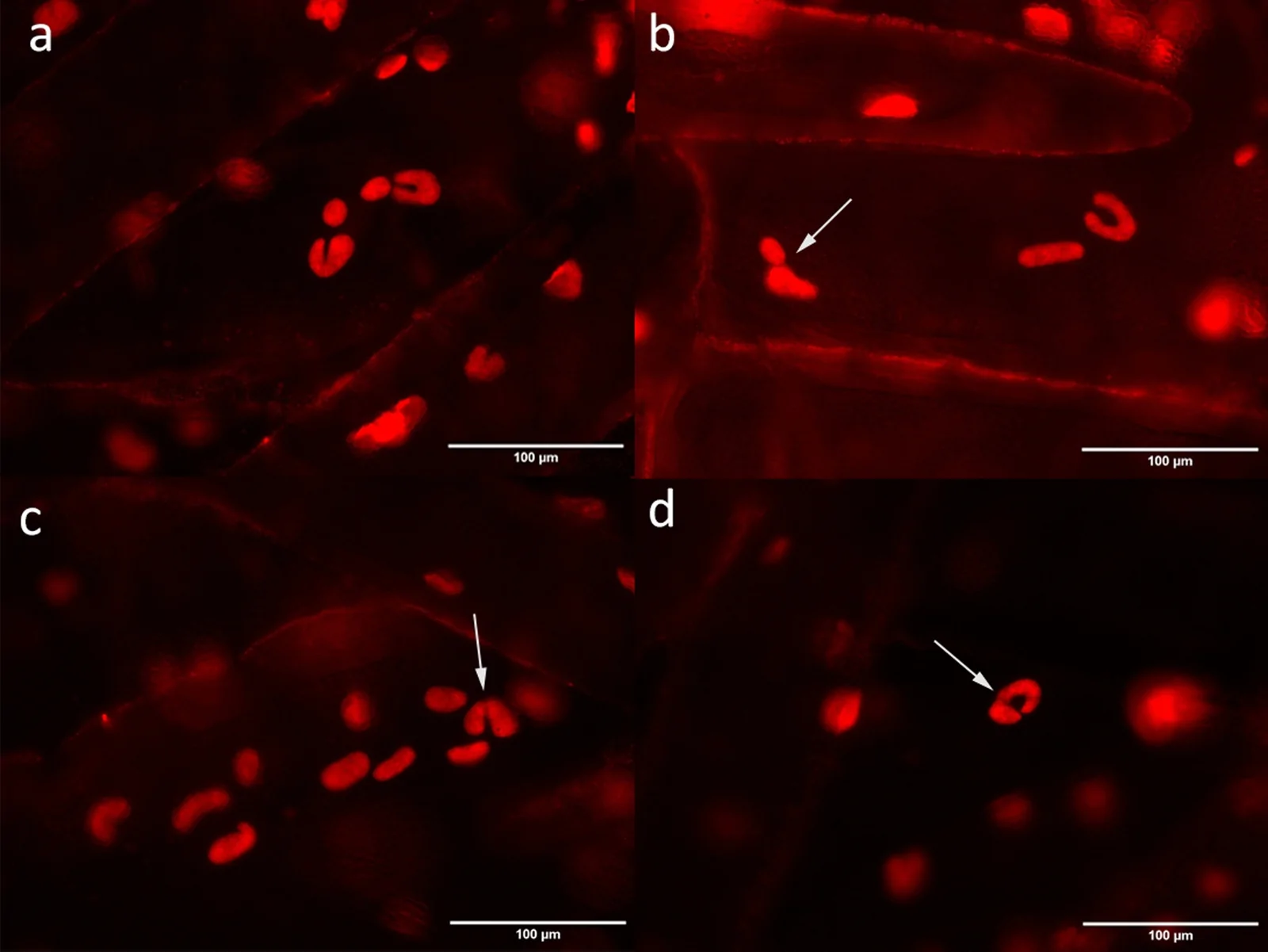
Fluorescence images of PI-stained nuclei in the apical part of *Chara hispida*. White arrows in **b**–**d** point at constrictions at nuclei

**Fig. 4:**
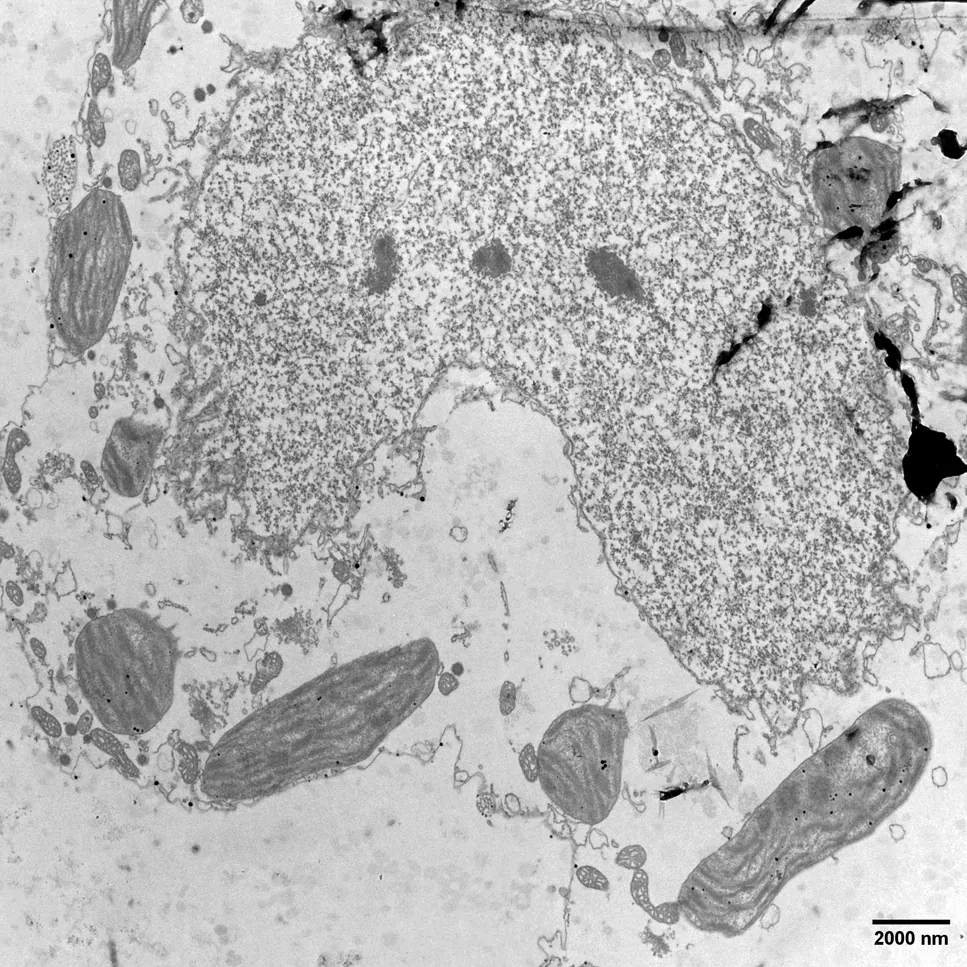
TEM image of a crescent-shaped nucleus in *Chara* sp

**Fig. 5:**
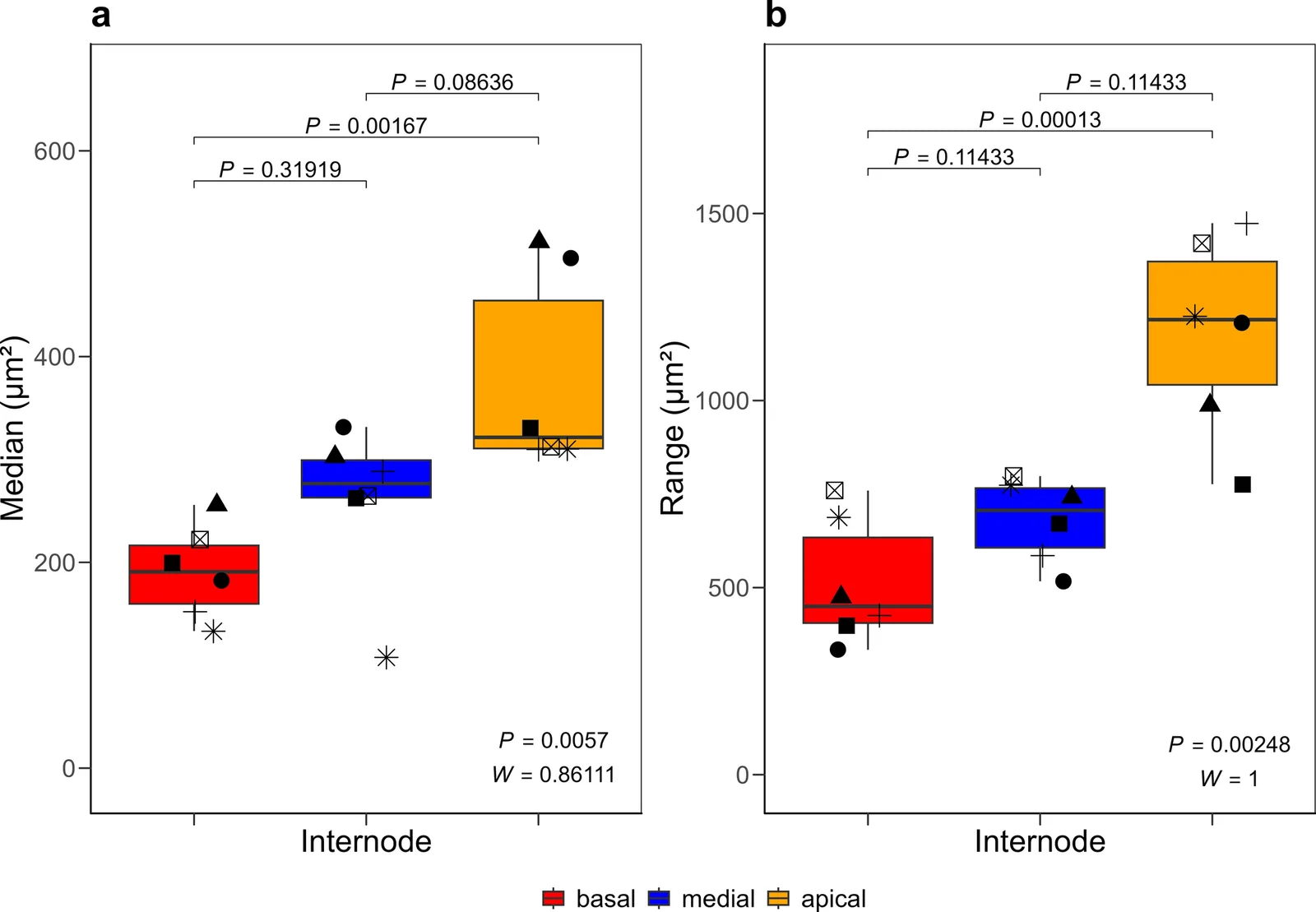
Nuclear cross-sectional area in *Chara hispida* across basal, medial and apical internodes (*n* = 6 plants per position; one image per plant per position; 50–100 nuclei summarized per image). **a** Median of nuclear cross-sectional area as an indicator of nuclear size. **b** Range of nuclear cross-sectional area as an indicator of size and shape diversity. The same point shape indicates the same replicate plant. *P* values from the Friedman rank-sum test and Kendall's *W* are shown in the bottom-right corner. *P* values above the boxplots indicate results of exact all-pairs comparisons of Friedman-type ranked data (Benjamini–Hochberg-adjusted)

PI-stained *Chara hispida* revealed a high density of nuclei in the apical region of the thallus, where multinucleation and diverse nuclear morphologies were observed (Fig. 2a–b). In contrast, the apical initial cell itself contained a single nucleus (Fig. 2a). Higher magnification of nuclei in the apical thallus region revealed shapes ranging from round and oval to elongated and crescent-shaped (Fig. 3). TEM imaging of a crescent-shaped nucleus in *Chara* sp. confirmed the nuclear identity of this nuclear morphology. The nucleus contained three nucleoli, and no obvious cytoskeletal or ER structures were visible within the limits of preservation of this section (Fig. 4). Some nuclei exhibited a constriction (Fig. 3b–d). In contrast, the basal region of the thallus showed a lower nuclear density, with nuclear sizes and shapes appearing more homogeneous (Fig. 2c, d). These qualitative observations were confirmed by quantitative analysis: both the median (as an indicator of nuclear size) (Fig. 5a) and the range of nuclear cross-sectional areas (as an indicator of size and shape diversity) (Fig. 5b) increased significantly from basal to apical internodes.

**Fig. 6:**
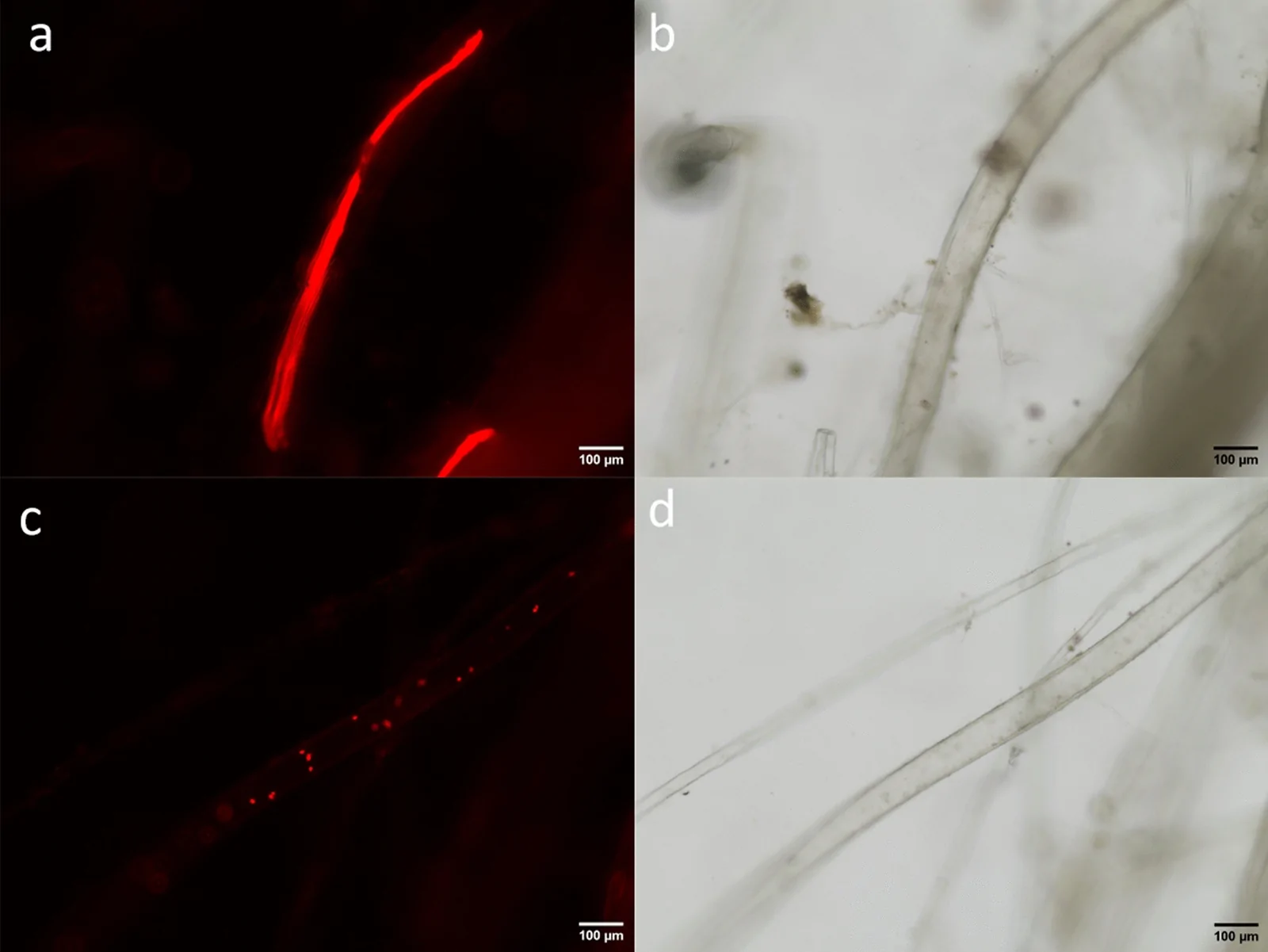
Fluorescence and light microscopic images of PI-stained rhizoids in *Chara hispida*. **a** Fluorescence image showing an elongated nuclear structure. **b** Light microscopic image of the same region. **c** Fluorescence image of small, rounded nuclei in a different rhizoid. **d** Corresponding light microscopic image

**Fig. 7:**
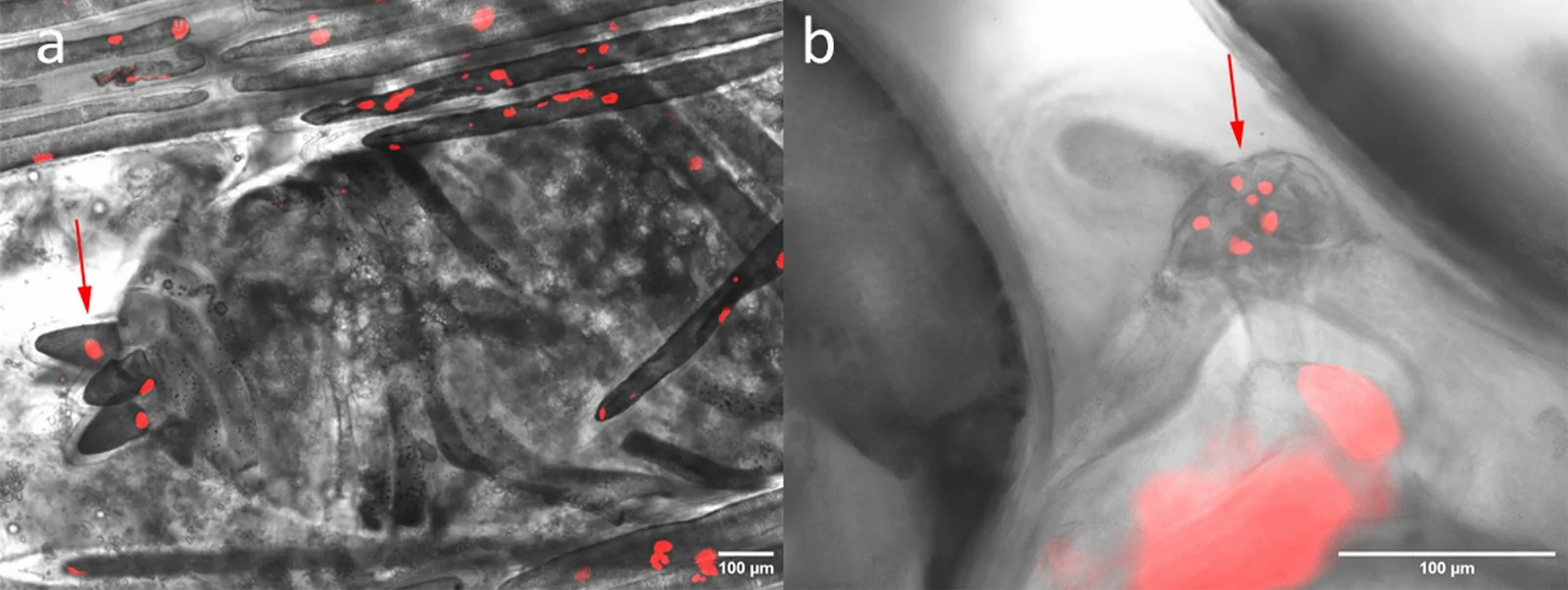
Merged fluorescence and light microscopic images of oogonia of two species stained with PI. Red arrows indicate the coronula. **a** *Chara hispida*. **b** *Tolypella nidifica*

**Fig. 8:**
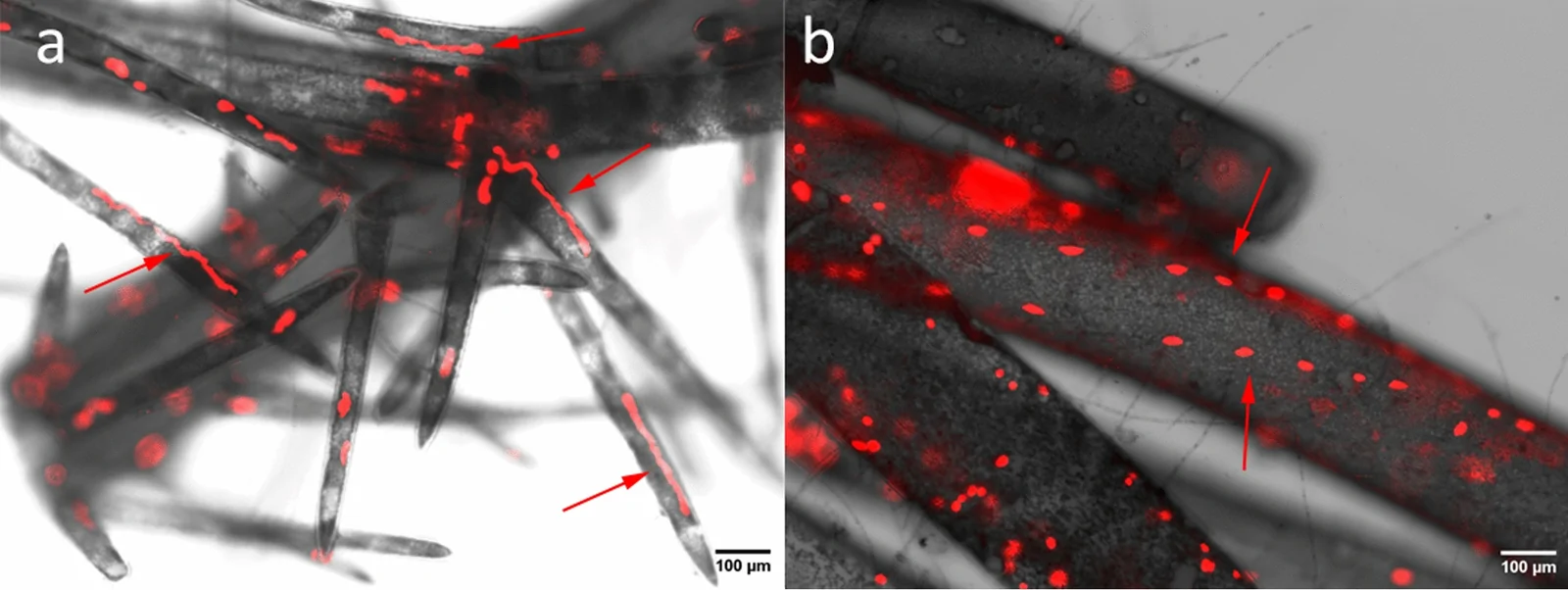
Merged fluorescence and light microscopic images of two species stained with PI. **a** Nuclei in *Chara aculeolata*; red arrows point at elongated nuclear structures in the spine cells. **b** Nuclei in *Sphaerochara canadensis*; red arrows point at spatially organized, evenly spaced nuclei pattern

**Fig. 9:**
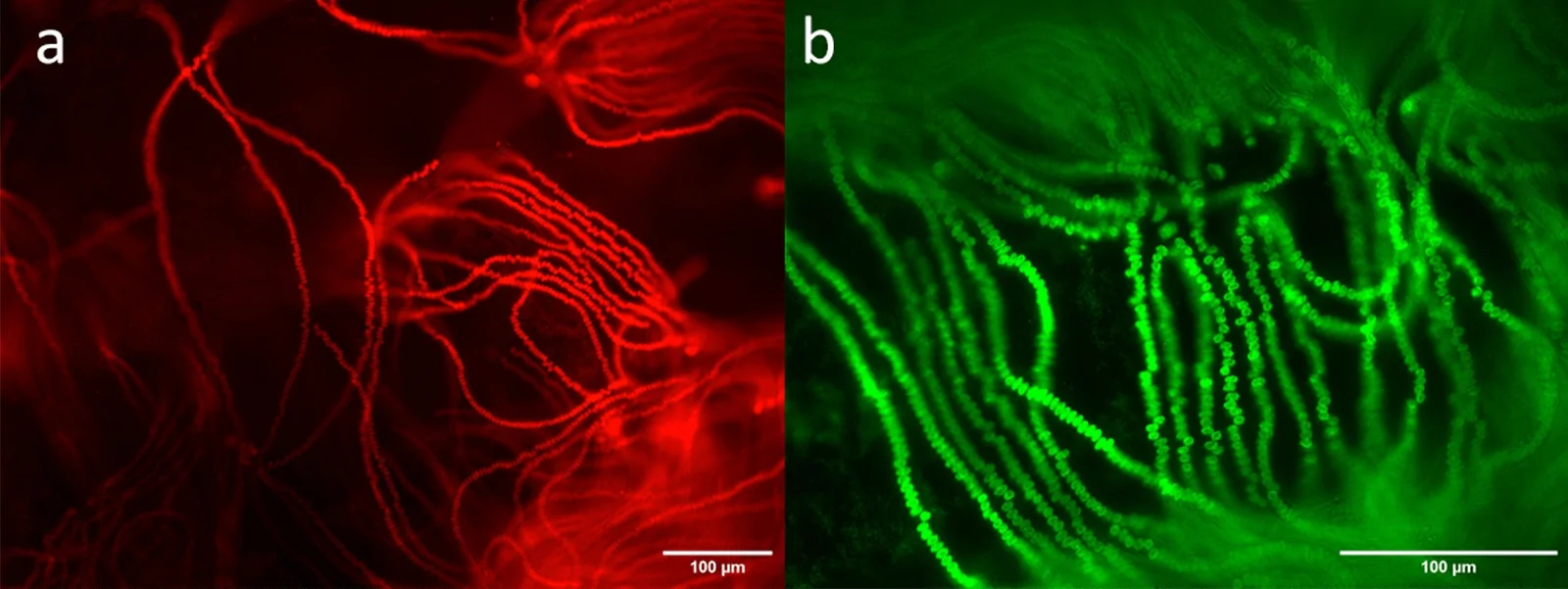
Fluorescence images of spermatogenous filaments in *Chara tomentosa* stained with PI (**a**) or stained with SYTOX Green (**b**)

Rhizoids of *Chara hispida* contained both small, rounded nuclei and elongated structures several hundred micrometres in length (Fig. 6a–d). In the coronula of *Chara hispida*, which is a crown-like structure at the apex of the oogonium, one nucleus per cell was observed (Fig. 7a). The same pattern occurred in *Tolypella nidifica* (O.F. Müll.) A. Braun (Fig. 7b) and *Nitella opaca* (C. Agardh ex Bruzelius) C. Agardh (Fig. S3b). Further analyses across different species revealed that only *Sphaerochara canadensis* (Sawa) Soulié-Märsche displayed nuclei in a spatially organized pattern (Fig. 8b). This phenomenon was not observed in any other studied ecorticated species (Fig. S3a, c, d, e). In *Chara aculeolata* Kütz., several hundred micrometres long nuclear structures were found in the spine cells (Fig. 8a). Antheridia of *Chara tomentosa*, in which the spermatogenous filaments contained densely packed nuclei of uniform shape and size, were readily stained with PI and SYTOX Green (Fig. 9a, b).

**Fig. 10:**
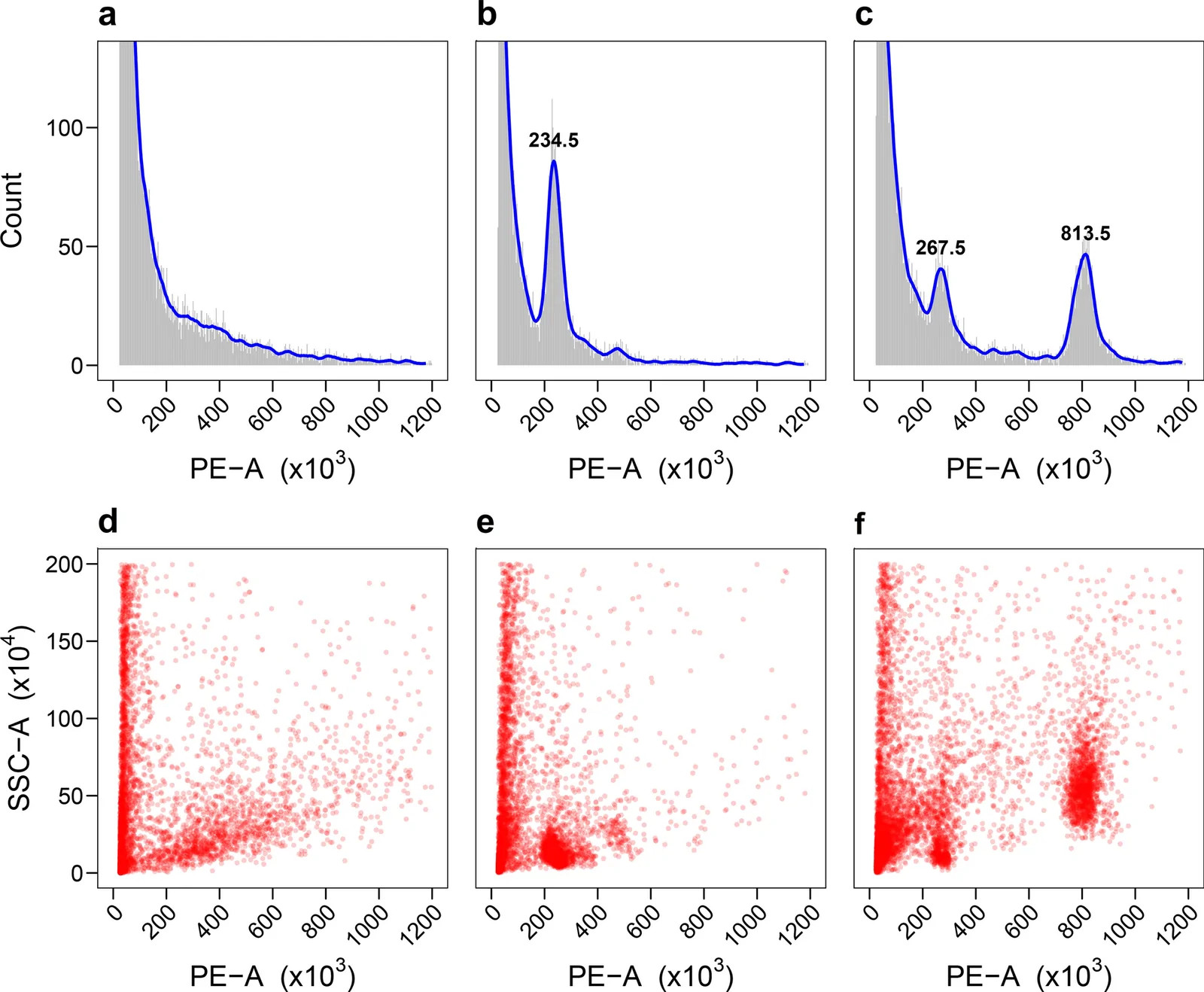
Histograms of relative nuclear DNA content and corresponding side-scatter (SSC-A) versus PE-A dot plots obtained by flow cytometry analysis of PI-stained nuclei isolated from different tissues. **a**, **d** Whole *Chara tomentosa* plant. **b**, **e** Antheridia of *Chara tomentosa*. **c**, **f** Antheridia of *Chara tomentosa* and leaves of *Secale cereale*

Flow cytometric analysis showed no distinct nuclear population for *Chara tomentosa* when whole thalli were analyzed (Fig. 10a, d). In contrast, a well-defined nuclear population was detected in the antheridia of *Chara tomentosa* at 234.5 × 10^3^ PE-A, i.e., the detector channel used for PI (Fig. 10b, e). After the addition of *Secale cereale* as an internal standard, the nuclear population of the antheridia was reproducibly detected at 267.5 × 10^3^ PE-A, and an additional peak appeared at 813.5 × 10^3^ PE-A, which corresponds to *Secale cereale* (Fig. 10c, f). Based on the standard, a genome size of 5.32 pg was calculated for the antheridia of *Chara tomentosa*.

## Discussion

This study optimized DNA staining and preparation techniques for visualizing nuclei in Charophyceae, with the goal of identifying a consistent cell type for genome size analysis via flow cytometry. It also investigates nuclear variation across cell types and species.

PI reliably stained nuclei across all examined tissues and produced minimal background, whereas DAPI and SYTOX Green labeled fewer nuclei and yielded more diffuse signal. The superior performance of PI is consistent with previous evaluations identifying it as a fluorochrome of choice for quantitative DNA staining in plants, owing to its largely sequence-independent intercalation into double-stranded DNA and its established use in flow cytometry (Doležel et al. 1992; Doležel and Bartos 2005; Sliwinska et al. 2022). DAPI, by contrast, binds preferentially to AT-rich regions of the minor groove of DNA and can therefore introduce bias related to genomic base composition (Barow and Meister 2002), which is particularly relevant for comparative quantification. SYTOX Green was originally developed as a viability indicator (Roth et al. 1997) and is widely used to label nuclei of non-viable cells in plant tissue (Truernit and Haseloff 2008). Its strong fluorescence enhancement upon nucleic-acid binding may have contributed to the diffuse background we observed. The highly pectic, polyanionic cell walls of Characeae (Sørensen et al. 2011; Domozych et al. 2012) may additionally have restricted dye penetration: although all three dyes used here carry a net positive charge, they differ in molecular size, lipophilicity and effective working concentration and may therefore experience different degrees of retardation by the cell wall. For routine visualization of nuclei and flow cytometry in Charophyceae, we therefore recommend PI.

Nuclear morphology was strikingly diverse, ranging from rounded and oval to elongated and crescent-shaped forms, consistent with earlier reports (Kaiser 1896; Shen 1967b). TEM imaging confirmed the crescent-shaped form as a nucleus, extending an earlier ultrastructural confirmation of round-to-oval forms in *Chara contraria* (Vouilloud et al. 2007). The ultrastructural preservation of our samples did not permit detailed claims about cytoplasmic elements adjacent to the nucleus, but no obvious cytoskeletal or ER structures were visible at the periphery of the crescent-shaped nucleus, consistent with the view that the shape is maintained by the nucleus itself rather than imposed by external mechanical constraints. In some nuclei, constrictions or indentations were apparent, which may reflect late stages of (a)mitotic division. For *Chara zeylanica* Willd., amitosis without a spindle apparatus has been proposed, involving symmetrical DNA distribution between daughter nuclei, indicating a regulated process (Shen 1967b). On the other hand, amitosis was also observed in *Chara contraria*, in which the amitotic division did not separate equivalent portions of the nucleus, as nuclear fragmentation resulted in the unequal segregation of chromatin (Vouilloud et al. 2007). Such unequal segregation is expected to generate considerable cell-to-cell heterogeneity in nuclear DNA content within a multinucleated thallus, and provides a plausible explanation for the broad, unstructured fluorescence profiles we recorded analyzing whole thalli by flow cytometry.

As shown for land plants, the shape of nuclei can vary by species, tissue, and cell type, and is regulated largely by nuclear envelope proteins (Goto et al. 2021). These proteins not only determine nuclear morphology but also contribute to essential cellular processes like gene expression, chromatin organization, signaling, and plant development. One physiological significance of nucleus deformation might be increasing the nuclear surface-to-volume ratio to facilitate Ca^2+^ signaling (Bootman et al. 2009) and enhancing messenger RNA export and translation near invaginations (Collings et al. 2000). For example, in *Arabidopsis* mutants, altered nuclear Ca^2+^ dynamics suggest a potential link between nuclear region structure and calcium signaling (Moser et al. 2020). Whether comparable functions drive the diversity of nuclear shape we observed in Characeae remains to be tested, but the parallels suggest that nuclear morphology in this group is unlikely to be purely passive.

*Chara hispida* displayed a striking gradient in nuclear cross-sectional area along the thallus axis, with significantly larger and more heterogeneous nuclear cross-sectional areas in apical regions when compared with the smaller, more uniform nuclei in basal internodes. As we measured nuclear size on 2D images, this is an indirect proxy for nuclear volume and therefore for DNA content. Comparable patterns have been described by Foissner and Wasteneys (2000) in other Characeae species. Shen (1967b) reported a similar progression in nuclear DNA content for *Chara zeylanica* from 2C in apical cells to 4C-32C in developing internodes, with a decline toward the most basal regions, using preparations that retained only the central cell with cortex removed. Despite this methodological difference, the broad pattern we describe is consistent with his observations.

Nuclear enlargement is frequently, though not exclusively, associated with increases in DNA content through endoreduplication (Sugimoto-Shirasu and Roberts 2003; Meier et al. 2016; Leitch and Dodsworth 2017). Endoreduplication, repeated rounds of DNA replication without intervening mitosis, is widespread in plant somatic tissues, can be induced by developmental cues, biotic interactions and environmental stress, and is commonly associated with enlarged cells and altered metabolic capacity (Leitch and Dodsworth 2017; Van de Peer et al. 2021). Because we did not measure DNA content at the single-cell level, endopolyploidy in basal to apical *Chara hispida* internodes remains a hypothesis rather than a demonstration in our data. It is important to keep this somatic endopolyploidy conceptually distinct from organism-level polyploidy resulting from whole-genome duplication in the germline (Van de Peer et al. 2021), a point we return to below.

In the rhizoids, both small, rounded nuclei and very large, elongated nuclei were observed, consistent with previous reports (Linsbauer 1927; Hasitschka-Jenschke 1960). Hasitschka-Jenschke (1960) hypothesized that rhythmic cell volume increases in *Chara contraria* indicate endomitotic polyploidization (endoreduplication), potentially up to 32C. This is paralleled by findings in land plant roots, where endoreduplication is associated with enhanced symbiotic efficiency, nutrient uptake, and rapid elongation (Van de Peer et al. 2021; Li et al. 2025).

We also observed species-specific differences: the spine cells of *Chara aculeolata* exhibited highly elongated nuclei. This distinct nuclear shape further supports the idea of endopolyploidy. Furthermore, multinucleated tissues were common across all examined species, but the spatial organization of nuclei varied. While nuclei generally appeared randomly distributed in both corticated and ecorticated species (see also Fig. S3), *Sphaerochara canadensis* exhibited a highly ordered, evenly spaced distribution pattern of nuclei. It is tempting to speculate that such a conspicuous arrangement of nuclei through the highly elongated internodal cells might facilitate uniform gene expression and maintenance of high growth rates. In rapidly growing root hairs, it has long been known that the actin and microtubule system positions the nucleus at a fixed distance behind the apical growth hotspot, and that altering this position leads to reduced growth rates and defects (Ketelaar et al. 2002; Brueggeman et al. 2022). Whether comparable cytoskeletal mechanisms organize the nuclear array of *Sphaerochara canadensis* remains an open and tractable question.

In addition to multinucleated structures, we observed mononucleated cells in specific reproductive and vegetative regions, namely in apical cells, the oogonial coronula, and the spermatogenous filaments of antheridia. These nuclei consistently appeared round to oval in shape, with smaller nuclei observed in the spermatogenous filaments. Of these, antheridia provided the most accessible source of a large, uniform population of nuclei for flow cytometry and yielded a clear nuclear peak in *Chara tomentosa*. With *Secale cereale* as an internal standard, we determined a genome size of 5.32 pg (1C) for *Chara tomentosa*. This value differs from the previously reported genome size of 7.4 pg (1C) for *Chara tomentosa* (f. *vulgaris*) (Maszewski and Kołodziejczyk 1991), but fits within the known range of genome sizes in the genus. According to the Kew Plant DNA C-values Database (Leitch et al. 2019) the smallest reported size is 1.92 pg (1C) for *Chara braunii* strain S276 (Nishiyama et al. 2018), while the largest is 19.6 pg (1C) for *Chara contraria* (f. *caespitosa*) (Maszewski and Kołodziejczyk 1991). The difference between our estimate and the earlier report may reflect methodological differences, the choice of standard, intraspecific variation, or population-level differences between sampling sites, possibilities that can only be resolved by repeated sampling incorporating a broader range of species from multiple geographic locations.

Our results are consistent with widespread endopolyploidy, as discussed earlier, but do not test for organism-level polyploidy. The two phenomena are often discussed together because both elevate nuclear DNA content, but they differ fundamentally in cause and consequences: endopolyploidy is a developmentally regulated, somatic process that can vary among cells within a single individual (Leitch and Dodsworth 2017), whereas polyploidy in the strict sense produces fixed, heritable changes across the organism and is a major driver of evolutionary diversification (Van de Peer et al. 2021). In Characeae, chromosome counts have been reported as multiples of a basic chromosome number (e.g., *n* = 14, 28, 42, 56 in *Chara*; Casanova 2015), which suggests that whole-genome duplication has indeed shaped the evolutionary history of the group. Importantly, Casanova (2015) demonstrated that chromosome counts can be obtained reliably from spermatogenous filaments, the same material that proved optimal for flow cytometry here, making antheridia an ideal target for combined cytometric and karyological analyses. Future work should extend genome size estimates across multiple species and populations of Characeae and combine flow cytometry with chromosome counts to distinguish endopolyploidy from true polyploidy.

In conclusion, we have shown that the nuclear system of Charophyceae exhibits a high morphological diversity within individuals and among species. We presented an easy-to-apply protocol for sample preparation allowing the analysis of nuclei via microscopy and flow cytometry. This will help to address both longstanding and emerging questions in Charophyceae biology, such as the site of meiosis, the mechanisms and functions of multinucleated cell formation and genome size evolution within Charophyceae.

## Supplementary Information

Below is the link to the electronic supplementary material.

Supplementary file1 (DOCX 2218 kb)

## Data Availability

Any data not included in the Supplementary Materials are available from the corresponding author upon request.

## Abbreviations

DAPI: 4′,6-diamidino-2-phenylindole
PE-A: Phycoerythrin area
PI: Propidium iodide
TEM: Transmission electron microscopy
