# Supplementary material for: Nuclear diversity in Charophyceae revealed by fluorescence staining and its implications for genome size estimation
Source: Planta. 2026 Aug 28;264(4):110. doi: 10.1007/s00425-026-05085-w (PMC13525061; doi:10.1007/s00425-026-05085-w)
Supplement: Supplementary file 1 [file 425_2026_5085_MOESM1_ESM.docx]

**Supplementary Material**

**Fig. S1** Overview of the thallus structure of *Chara hispida*. The left panel was assembled from multiple single images using Adobe Photoshop Lightroom 6. The schematic overview was created with BioRender (<https://BioRender.com/d6x2jt8>)*.*

**Fig. S2** *Chara hispida* stained with three fluorescent dyes. **a** PI. **b** Sytox Green. **c** DAPI.

**Fig. S3** Nuclei in different Characeae species. **a** *Nitella opaca*. **b** *Nitella opaca*. **c** *Lamprothamnium papulosum*. **d** *Chara braunii*. **e** *Chara braunii*. **f** *Chara papillosa*.
